# Dynamics of dengue and SARS-COV-2 co-infection in an endemic area of Colombia

**DOI:** 10.1186/s40794-022-00169-3

**Published:** 2022-05-15

**Authors:** Tomás Acosta-Pérez, Tomás Rodríguez-Yánez, Amilkar Almanza-Hurtado, María Cristina Martínez-Ávila, Carmelo Dueñas-Castell

**Affiliations:** 1 Internal Medicine, Universidad Libre, Cali, Colombia; 2 Intensive Care Unit, Gestión Salud IPS, Cartagena, Colombia; 3 Department of Epidemiology and Clinical Research, BIOTOXAM GROUP, Universidad de los Andes, Cartagena, Colombia

**Keywords:** COVID-19, SARS-CoV-2, Coinfection, Dengue, Colombia, Overlap disease

## Abstract

Since the COVID-19 outbreak, millions of people have been infected with SARS-CoV-2 around the world. An area of epidemiological relevance is Latin America, tropical regions, due to the distribution of endemic diseases such as chikungunya, dengue (DENV), malaria, Zika virus, where febrile disease abounds. The early signs and symptoms of DENV and COVID-19 could be similar, making it a risk that patients may be wrongly diagnosed early during the disease. The problem increases since COVID-19 infection can lead to false positives in DENV screening tests. We present two cases of acute undifferentiated febrile syndrome that were diagnosed with SARS-CoV-2 and DENV co-infection, confirmed by ELISA and RT-PCR for both viral pathogens. The occurrence of simultaneous or overlapped infections can alter the usual clinical course, severity, or outcome of each infection. Therefore, epidemiological surveillance and intensified preparation for those scenarios must be considered, as well as further studies should be done to address cases of co-infection promptly to avoid major complications and fatal outcomes during the current pandemic. Other endemic tropical diseases should not be neglected.

## Introduction

Severe acute respiratory syndrome coronavirus-2 (SARS-CoV-2) causing coronavirus disease 2019 (COVID-19) has spread rapidly throughout Latin America. In Colombia and other tropical countries, the pandemic potentially coincides with another epidemic already in the region, dengue (DENV) [1]. DENV is an arboviral infection transmitted by the *Aedes aegypti* mosquito characterized by acute onset of high fever [2], meanwhile, COVID-19 is a viral infection that usually begins with respiratory symptoms. There are similarities in the initial presentation of patients with COVID-19 and dengue [3], headache, myalgia, fever, associated with leukopenia, thrombocytopenia, abnormal liver function tests and other laboratory findings [4].

In tropical countries, COVID-19 can easily be misdiagnosed with dengue or other more common infectious diseases, leading to a delay in the diagnosis of COVID-19 infection and further spread of the virus [3]. Failure to consider COVID-19 in such cases has serious implications for the patient, as well as public health. Public health concerns are generated due to the possibility that the presence of both viruses and the development of co-infections harm mortality and other clinical outcomes [5]. Co-infection can be defined as the simultaneous presence of two or more infections, which can increase the severity and duration of one or both diseases [3].

Information about DENV and COVID-19 co-infection is scarce, and the dynamics of the disease and outcomes may be altered in this scenario. Rapid serological testing for dengue sometimes gives a false positive in acute undifferentiated febrile disease in the COVID-19 infection setting [4]. This situation complicates things further, thus, it can be difficult to distinguish early infections vs. co-infection, generating a significant risk for the population and demanding greater attention for healthcare systems.

In this paper, we describe 2 patients with co-infection of SARS-CoV-2 and DENV, confirmed by ELISA and RT-PCR for both viral pathogens in Colombia, to disclose important details of this emerging overlapping coinfection. This study has important implications for distinguishing and determining co-infection from mono-infection, as well as the clinical picture in such cases of co-infection between DENV and SARS-CoV-2.

## Methods

This study included two cases of COVID-19 co-infection with DENV admitted in an intensive care unit (ICU) of a third level hospital of an endemic country (Cartagena, Colombia) during COVID-19 pandemic. Clinical and laboratory investigations that were undertaken to determine the diagnosis included: images: x-ray or CT, serum chemistry, inflammatory biomarkers, molecular test.

## Results: cases presentations

### Case 1

A 65-year-old Colombian woman with a history of hypertension, presented with 8 days of asthenia, retroorbital pain, frontoparietal headache in location, rated 6/10, joint and muscle pain. 2 days before admission, he added dry cough, sore throat, and sensation of dyspnea without any other associated symptoms. She was observing quarantine and denied contact with cases suspicious or confirmed of COVID-19 infection as well as mosquito bites. The patient had self-medicated with paracetamol, which provided temporary relief; however, her condition was persistent, prompting consultation.

Her vital signs revealed a body temperature of 38.9 °C, a respiratory rate of 26 breaths/minute, a pulse rate of 110/minute, and a blood pressure of 130/80 mmHg. Oxygen saturation at presentation was 90% in room air. Physical examination only showed bibasilar crackles and a petechial rash. Analysis revealed leukopenia with lymphopenia, thrombocytopenia, moderate D-Dimer, transaminases, C-reactive protein (CRP), and elevation of LDH (Table 1).

**Table 1:** Timeline events of DENV and SARS-CoV-2 coinfection cases

	Case 1	Case 2	Reference value
Days of symptoms on admission	8	3	
Admission diagnosis	COVID-19	COVID-19	
Comorbidities	Arterial Hypertension	None known	
Day 1 symptoms	Headache, myalgia, altralgia, fever	Fever, dry cough, asthenia, adynamia	
Respiratory symptoms	48 hours after initial symptoms	From the 1st day of the onset of symptoms	
Day 1 hospitalization	Suspicion and sampling NS1/IgM DENV	Suspicion and sampling RT-PCR COVID-19	
Leukocytes/mm^3^	3100	2180	4000–11,000
Lymphocytes/mm^3^	410 (10%)	296.48 (13,6%)	20–40%
Hemoglobin (mg/dL)	13	11.8	13–15
Hematocrit (%)	48	45	30–45
Platelet/mm^3^	47,000	70,000	150,000–450,000
AST (U/L)	48	64	< 40
ALT (U/L)	25	33	< 40
D-Dimer (ng/mL)	639	1475	< 500
CRP (mg/dL)	9	49.8	< 1
LDH (U/L)	494	989	< 150
Serum creatinine (mg/dL)	0.75	1.9	0.5–1
Day 3	NS1/IgM positive (9 days of symptoms)	RT-PCR positive for COVID 19	
Day 4	RT-PCR positive for COVID 19	Persistent thrombocytopenia suspected DENV ELISA and collection of RT-PCR sample collection	
Day 5	RT–PCR DENV positive for DENV serotype 2	ELISA positive for DENV. RT–PCR DENV positive for DENV serotype 3	
Clinical course and outcome	Patient progressed to ventilatory failure requiring prolonged invasive mechanical ventilation, needed for tracheostomy, transferred to a chronic care center	Patient with acute ventilatory failure, requiring invasive mechanical ventilation, with progressive clinical deterioration and multiple organ failure, that eventually led to death	
Final diagnosis	DENV2 and SARS-CoV-2 co-infection	DENV3 and SARS-CoV-2 co-infection	

**Fig. 1:**
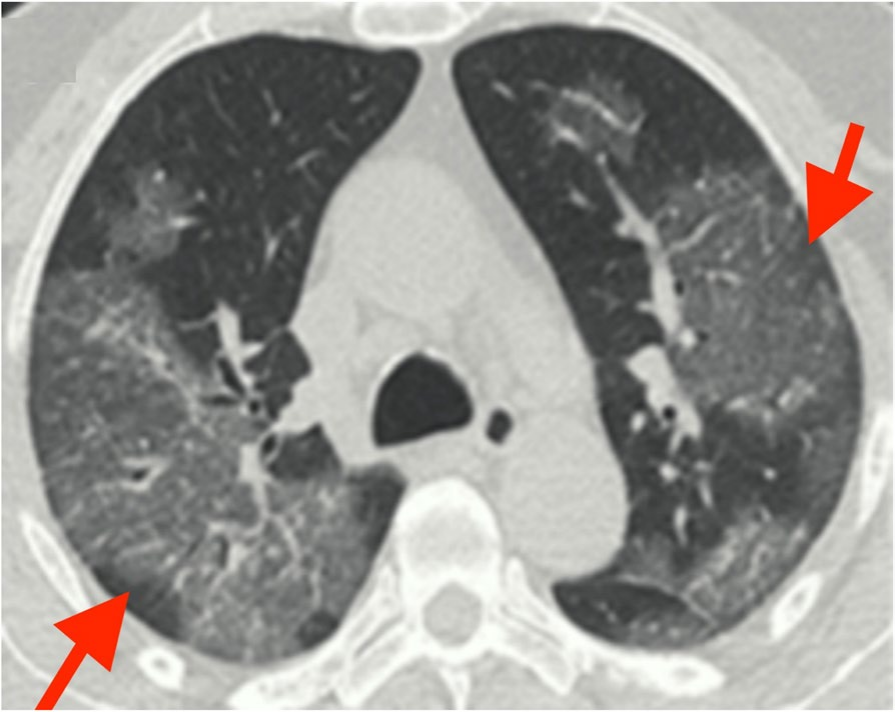
Axial CT scan view showing scattered ground glass in both lung fields, with 50% lung involvement (red arrows)

A chest CT scan was performed, and scattered ground glass images were shown in both lung fields, compromising 50–60% of the lung parenchyma due to probable viral pneumonia (Fig. 1).

She was hospitalized with supportive treatment, dexamethasone (after the recovery trial), IV fluids, paracetamol, additional oxygen with nasal cannula, and close monitoring. The rapid dengue test revealed a positive nonstructural protein 1 (NS1) with positive immunoglobulin (Ig) M and IgG and a nasopharyngeal swab for SARS-CoV-2 real-time reverse transcriptase (RT-PCR) was taken.

This treatment did not improve her symptoms and has gradually worsened. ABG tests were performed which showed severe hypoxemia (partial pressure of oxygen [PaO2]: 36 mmHg, PaO2/fraction of inspired oxygen [FiO2] 68 mmHg. A repeat of the complete blood count showed a sudden drop in the platelet count to 20,000/mm^3^ without any visible bleeding. Therefore, dengue RT-PCR was requested due to doubtful diagnosis and DENV serotype 2 (DENV2) was detected. RT–PCR for SARS-CoV-2 was positive, confirming the diagnosis of dengue with warning signs associated with severe COVID-19.

She was transferred to the ICU, for ventilatory support due to progression to acute respiratory distress syndrome and refractory hypoxemia that requires invasive mechanical ventilation. Clinical characteristics were attributed to SARS-CoV-2 infection. On subsequent days, increasing trends in the number of platelets and leukocytes were observed, and clinical symptoms improved. However, extubation was not achieved; she required a tracheostomy and was discharged to a chronic care unit for pulmonary rehabilitation.

### Case 2

58-year-old Colombian male, without known medical history, complained of persistent fever of 39 °C, diarrhea, dyspnea, asthenia, myalgias, and dry cough that lasted 3 days; he had tested positive for SARS-COV 19 by RT-PCR. Due to the worsening of cough, dyspnea, and shortness of breath, he consulted an online clinic where he was referred to the hospital for evaluation. On examination, he appeared dehydrated, with peripheral cyanosis, somnolent but arousable with marked respiratory effort and bibasilar crackles. Vital signs with a pulse rate of 108/minute, respiratory rate of 32 breaths/minute, blood pressure of 100/70 mmHg. Oxygen saturation at presentation was 84% in room air, without any other findings on physical examination.

**Fig. 2:**
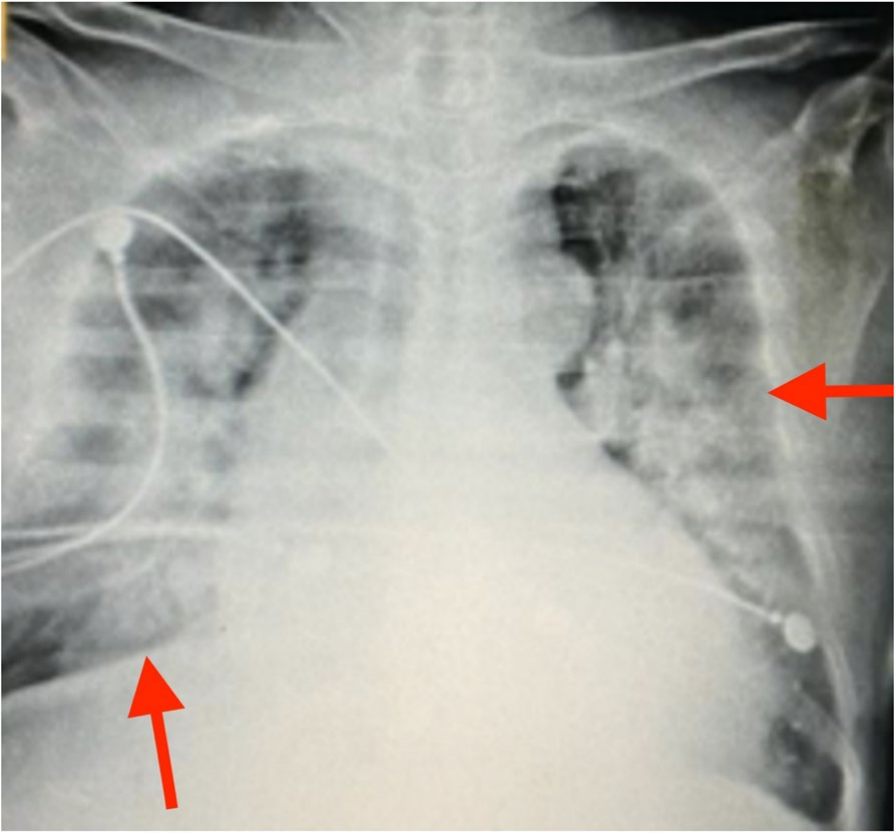
Portable chest radiograph showing multiple radiopacities of interstitial occupation and peripheral distribution (red arrows)

Immediately, a portable chest radiograph was performed showing multiple radiopacities of interstitial occupation and peripheral distribution (Fig. 2). Considering that he has acute respiratory failure supported by clinical findings (tachypnea, tachycardia, cyanosis, altered levels of consciousness, diffuse crackles and respiratory effort), he was intubated (pressure control ventilation [PCV] mode, inspiratory oxygen fraction [FiO_2_], 0.5, positive end-expiratory pressure [PEEP], 10 cmH_2_O; inspiratory pressure [Pi], 15 cmH_2_O; inspiratory time [Ti], 1.5 s; frequency [f], 12 per minute) and transferred to the ICU.

Arterial blood gas analysis revealed a pH of 7.45, an oxygen pressure of 48 mmHg, a carbon dioxide pressure of 30 mmHg, and a bicarbonate of 21.1 mmol/L, a PaO2/fraction of inspired oxygen [FiO2] 96 mmHg. Laboratory tests had leukopenia with lymphocytopenia and thrombocytopenia. Renal function, liver enzymes, CRP, serum LDH, and D-dimer were elevated (Table 1). Due to severe thrombocytopenia in an endemic area, dengue serology was positive for NS1 antigen. To confirm the diagnosis, an anti-dengue IgM/IgG ELISA, serology test and RT-PCR were requested and DENV serotype 3 (DENV3) was detected. Therefore, the patient was diagnosed with severe COVID-19 with dengue fever with signs of red flags.

During his hospitalization, the patient’s acute hypoxic respiratory failure did not recover, his oxygenation was poor, despite the tracheal intubation connected to the ventilator. Renal and liver function continued to decline. Subsequently, he became hypotensive and started norepinephrine for suspected cardiogenic vs septic shock. Empiric treatment with broad-spectrum antibiotics, dexamethasone (after recovery trial), and IV fluids was started. During this time, the ICU-prone ventilation protocol was initiated to improve oxygenation to his lungs.

The patient’s condition deteriorated sharply, developing multiorgan failure, characterized by pulmonary, renal, liver, and possible neurologic compromise. The patient remained on life-sustaining support. After 17 days in the ICU, Extracorporeal membrane oxygenation (ECMO) was performed. Despite the best efforts of the medical staff, the patient eventually died.

## Discussion

The causes of acute febrile illness (AFI) in Colombia are diverse [6], including multiple arboviral infections such as: chikungunya, zika, and DENV, which is the most frequently reported [2]. However, in the epidemiological context of the COVID-19 pandemic, the possibility of the occurrence of these diseases should not be underestimated. In the cases described, it was pertinent to rule out infection by DENV and COVID-19. COVID-19 and DENV infections are difficult to distinguish, as they share clinical and laboratory characteristics, which can lead to misdiagnosis or delayed treatment and patient isolation [3, 7].

Different studies have raised the possible cross-reaction between DENV and SARS-CoV-2 antibodies, which generates a risk of false positives and diagnostic doubts [7, 8]. Information related to infection by one or another agent is available; to date, the effects of coinfection remain unknown [9]. The cases of co-infection by SARS-CoV-2 and influenza do not appear to have a more severe course, but they showed similar clinical characteristics to patients affected in isolation by COVID-19 [10]. Cases of coinfection of SARS-CoV-2 with microorganisms such as *Mycoplasma pneumoniae, influenza virus, cytomegalovirus,* HIV*, Legionella, Pneumocystis jirovecii,* and even with multiple respiratory viruses have been reported [11]. In endemic areas such as Colombia, establishing the presence of multiple agents is essential to carry out an adequate therapeutic approach to avoid complications and fatal outcomes.

Recent studies suggest that COVID-19 and DENV coinfection presents less severe symptoms compared to isolated monoinfection, probably associated with opposite pro-and anticoagulant states triggered by SARS-CoV-2 and DENV respectively [12]. Furthermore, the possible improvement of DENV has been considered when there is a second infection with different viruses has been considered [13]. In this article, we present two cases of severe coinfection with hematocrit and ventilatory compromise, one of which had a fatal outcome.

The clinical and laboratory manifestations of each entity can have considerable overlap, presenting with fever, myalgia, asthenia, adynamia, diarrheal episodes, and dermatological lesions (Table 2) that make the differential diagnosis difficult [5]. Studies in Latin America have revealed that both viral diseases can trigger secondary hemophagocytic lymphohistiocytosis [6].

**Table 2:** Comparisons and differences of COVID-19 and DENV

Symptoms and laboratory findings	COVID-19	DENV
Fever	+++	+++
Headache	++	+++
Retro-orbital pain		++
Asthenia	+	++
Rash	+	++
Purpura		++
Myalgia/Arthralgia	+	++
Dyspnea	++	+
Anorexy	+	+
Cough	+++	+
Chest pain	++	+
Pharyngitis	++	++
Anosmia, eugesia	+++	+
Diarrhea	+	+
Nausea, emesis	+	+
Abdominal pain		++
Neurological Agitation/Alteration	+	+
CRP	++	
Lymphocytes	↓↓	↓
Neutrophils	↑	↓
Platelets	↓	↓
Ferritin	↑	↑
Transaminases	↑	↑
D-Dimer	↑	

Legend: + stands for frequency of findings, more than 1 (+) corresponds to frequently. ↑ elevated levels, decreased levels. Adapted from: [5]

In relation to laboratory tests, when having a patient with AFI, at least a complete blood count should be performed, liver enzymes, CRP, and kidney function should be performed [6]. In COVID-19, the most common hematologic abnormality is lymphopenia, present in approximately 80% of individuals, neutrophils are often elevated, and white blood cell count may be normal [14]. In DENV, the hematological parameter of interest is hemoconcentration, which represents an alarm sign [2, 15]; also, unlike COVID-19, thrombocytopenia and neutropenia can occur. The association between leukopenia and thrombocytopenia occurs in both entities, being more frequent in DENV than in COVID-19, occurring in 60–80% of cases [5, 15]. Liver function is also usually affected, with an increase in aminotransferases (AST and ALT) by 25 and 33%, respectively; as well as an increase in CRP in up to 60% of individuals [2, 15].

It is important to know and properly interpret the laboratory analysis, as according to the chronology and clinical course of the infection, alterations that represent some alarm sign (hemoconcentration in DENV), severity, or prognosis (D-dimer in COVID-19) may occur. Likewise, there are tests, such as ferritin, which are biomarkers of interest in COVID-19; but even though it can also be increased in DENV, they are not clinically important [9]. In our case, both patients presented thrombocytopenia, elevated transaminases, and increased CRP, making differentiation difficult from the clinical approach point of view.

Within the differential diagnoses, oriented by respiratory symptoms, common respiratory pathogens such as *Streptococcus* spp. *and Mycoplasma pneumoniae*, influenza, emerging diseases such as leptospirosis or toxoplasmosis are included. Up to 25% of patients with DENV present with respiratory symptoms [1, 3, 4]. The clinical presentations similar to DENV can be caused by other arboviruses such as chikungunya virus presented in early 2016 and Zika virus in 2017, malaria, Q fever, leptospirosis, salmonellosis, and primary HIV infection depending on the prevailing clinical context [6]. Considering the current pandemic, in the cases described, no viral panel was carried out for other respiratory viruses or arboviruses, as the certainty of clinical evidence leaned toward COVID-19.

In fact, in an Argentine retrospective study, they suggest that the existence of prolonged fevers longer than 10 days, headache, rash, whether there are respiratory symptoms, should lead to the suspicion of a concomitant COVID-19 and DENV infection; respecting the performance of adequate confirmatory tests, since rapid serological tests for DENV can cross-react with SARS-CoV-2 antigens and give false positives [12]. A systematic review of 15,976 samples indicates that the use of antibody tests for COVID-19, in particular rapid test employing lateral flow immunoassays, have limited benefits in the point-of-care testing [16], particularly in the early phase of SARS-CoV-2 infection leading to a significant hurdle to rely on the laboratory diagnosis [17]. It is worth to mention that a Peruvian cohort, the largest in Latin America, affirms that possible cross-reactions of IgM/IgG-DENV rapid test results concerning antibodies developed after infection with SARS-CoV-2 are not ruled out when describing cases of false positives in rapid tests DENV [18]. However, in this cohort they stated that there would be no cross-reaction between the rapid test of the NS1-DENV antigen and the IgM and IgG antibodies of SARS-CoV-2 since the patients hospitalized with COVID-19 days later presented an unfavorable and unusual evolution after detecting the presence of the viral agent of the vector *Aedes aegypti* [18]. Furthermore, according to the Colombian consensus on the care, diagnosis, and treatment of SARS-CoV-2 infection, positivity in ELISA and molecular tests such as RT-PCR confirmed co-infection with SARS-CoV-2 and DENV. Taking into account the following information, our cases coincide with what is stated in the literature.

## Conclusions

The medical challenge of co-infection of SARS-CoV-2 and DENV lies in the similarity of the clinical and laboratory characteristics of the two infections. The shared pathophysiology and endotheliotropic nature of both viruses could condition an amplified immune response in the host, causing the clinical presentation to overlap. It is necessary to carry out adequate clinical reasoning, remembering the possibility of COVID-19 in patients DENV positive and vice versa, since there could be cross reactions in laboratory tests. To avoid misdiagnosis or delayed treatment and patient isolation, we recommend asking for confirmatory tests when there are doubts; considering the result will affect treatment, prognosis, and outcome. Prospective studies are needed to allow us to understand the behavior and dynamics of this association and to identify the impact in terms of morbidity and mortality during co-infection.

## Data Availability

We have presented the data of the patients in the manuscript as tables and have submitted the figures separately as figures.
